# The Epistatic Relationship between BRCA2 and the Other RAD51 Mediators in Homologous Recombination

**DOI:** 10.1371/journal.pgen.1002148

**Published:** 2011-07-14

**Authors:** Yong Qing, Mitsuyoshi Yamazoe, Kouji Hirota, Donniphat Dejsuphong, Wataru Sakai, Kimiyo N. Yamamoto, Douglas K. Bishop, XiaoHua Wu, Shunichi Takeda

**Affiliations:** 1 Department of Radiation Genetics, Graduate School of Medicine, Kyoto University, Kyoto, Japan; 2 Department of Radiation and Cellular Oncology, University of Chicago, Chicago, Illinois, United States of America; 3 State Key Laboratory of Biotherapy and Cancer Center, West China Hospital, West China Medicinal School, Sichuan University, Chengdu, China; National Cancer Institute, United States of America

## Abstract

RAD51 recombinase polymerizes at the site of double-strand breaks (DSBs) where it performs DSB repair. The loss of RAD51 causes extensive chromosomal breaks, leading to apoptosis. The polymerization of RAD51 is regulated by a number of RAD51 mediators, such as BRCA1, BRCA2, RAD52, SFR1, SWS1, and the five RAD51 paralogs, including XRCC3. We here show that *brca2-null* mutant cells were able to proliferate, indicating that RAD51 can perform DSB repair in the absence of BRCA2. We disrupted the *BRCA1*, *RAD52*, *SFR1*, *SWS1*, and *XRCC3* genes in the *brca2-null* cells. All the resulting double-mutant cells displayed a phenotype that was very similar to that of the *brca2-null* cells. We suggest that BRCA2 might thus serve as a platform to recruit various RAD51 mediators at the appropriate position at the DNA–damage site.

## Author Summary

Mutations in BRCA1 and BRCA2 predispose hereditary breast and ovarian cancer. Such mutations sensitize to chemotherapeutic agents, including camptothecin, cisplatin, and poly(ADP-ribose) polymerase (PARP) inhibitor, since RAD51 mediators including both BRCA proteins promote repair of DNA lesions induced by these drugs. Little is known of the functional relationships among RAD51, BRCA2, and other RAD51 mediators, because no *brca2-null* cells were available. Furthermore, the phenotype of *sws1* mutants has not been documented. We here disrupted every known RAD51 mediator and analyzed the phenotype of the resulting mutants in both BRCA2-deficient and -proficient backgrounds. The understanding of the function of individual RAD51 mediators and their functional interactions will contribute to the accurate prediction of anti-cancer therapy efficacy.

## Introduction

Homologous recombination (HR) maintains genome integrity by accurately repairing double-strand breaks (DSBs) that arise during the mitotic cell cycle or are induced by radiotherapy [1], [2]. HR also plays an important role in releasing the replication forks that stall at damaged template DNA strands [3], [4]. Thus, effective HR makes tumor cells tolerant to the chemotherapeutic agents that damage DNA and stall replicative DNA polymerases. Such chemotherapeutic agents include cis-diaminedichloroplatinum(II) (cisplatin), camptothecin, and poly(ADP-ribose) polymerase (PARP) inhibitors, including olaparib (AstraZeneca). Cisplatin is a crosslinking agent that generates intra- and inter-strand crosslinks and thereby stalls replicative DNA polymerases. Camptothecin inhibits the ligation of single-strand breaks (SSBs) that are formed during the normal functioning of topoisomerase 1. Resulting unrepaired SSBs are converted to DSBs upon replication. Similarly, PARP inhibitors interfere with SSB repair [5]. Since HR plays a major role in repairing DNA lesions generated by camptothecin, cisplatin, and PARP inhibitors [6], measuring HR efficiency in individual malignant tumors may help predict the efficacy of these chemotherapeutic treatment for each tumor [7]–[9].

HR-dependent DSB repair is accomplished by the following step-wise reactions [10]. DSBs are processed by the Mre11/Rad50/Nbs1 complex and the CtIP, Exo1 and DNA2 nucleases to develop 3′ single-strand DNA (ssDNA) tails [11]–[17]. RAD51, an essential recombinase, polymerizes on these ssDNAs, leading to the formation of nucleoprotein filaments. These filaments undergo homology search and subsequent invasion into homologous duplex DNA to form a D-loop structure, where they serve as a primer for DNA synthesis [18], [19]. After the extended end is displaced from the D-loop, it anneals to its partner-end to complete DSB repair. We know that RAD51 plays a key role in HR in vertebrate cells, as inactivation of RAD51 results in the accumulation of chromosomal breaks in mitotic cells and inhibits the completion of even a single cell cycle [1], [2]. The polymerization of RAD51 at damage sites is strictly regulated by a number of accessory factors (hereafter called RAD51 mediators), including the five RAD51 paralogs, SWS1, RAD52, SFR1, BRCA1, BRCA2, and PALB2 [3], [20]–[27]. The functional relationships of these RAD51 mediators are poorly understood, because cells deficient in multiple RAD51 mediators have not been established.

BRCA2 was originally identified as a tumor suppressor, as germline mutation of the *BRCA2* gene results in a high risk of developing breast, ovarian, pancreatic, prostatic, and male breast cancer [3], [20], [28], [29]. BRCA2 is recruited to processed DSBs, and facilitates the assembly of RAD51 at the single-strand tail. The middle of the BRCA2 protein has eight BRC repeats, comprising 26 amino acids. Biochemical studies have revealed that individual BRC repeats prompt the loading of RAD51 on ssDNA [30], [31]. Since no *brca2-null* cells have been established, the function of BRCA2 has been postulated from the phenotypic analysis of mice carrying an allele, extending from the N-terminus to the BRC3 motif (hereafter *brca2tr*) that encodes a truncated form of BRCA2. Cells derived from *brca2tr* mice and *brca2tr* DT40 cells are able to proliferate and exhibit increased sensitivity to ionizing radiation, camptothecin, and cisplatin [32], [33]. It remains unclear whether *brca2 null* cells display the same phenotype as do *rad51* cells, or a milder phenotype.

The roles of the RAD51 mediators have been characterized by phenotypic analysis of their mutants. Mammalian *brca1*-deficient cells show normal focus formation of the RPA ssDNA binding protein but diminished RAD51 focus formation at DSBs, indicating that BRCA1 facilitates the polymerization of RAD51 after the resection of DSBs [15], [34]. DT40 cells deficient in any one of the five RAD51 paralogs show a very similar phenotype, including compromised RAD51 focus formation and the same degree of DNA damage sensitivity [35], [36], suggesting that these five proteins form a functional unit in the promotion of RAD51 polymerization in which each RAD51 paralog is essential for its function. No biochemical studies have yet defined the molecular mechanisms underlying the promotion of RAD51 assembly by BRCA1, the RAD51 paralogs, or SWS1. SWS1 is another RAD51 mediator [26], and *sws1*-deficient vertebrate cells have not yet been reported. SFR1 was originally identified in fission yeast [25], and its role in *in vitro* HR reactions [37], [38] as well as the phenotypic analysis of *sfr1*-deficient mice have been recently reported [39]. The biochemical character of full-length human BRCA2 has been recently documented, whereas no biochemical studies have defined its functional interaction with other RAD51 mediators or the molecular mechanisms underlying the promotion of RAD51 assembly by BRCA1, the RAD51 paralogs, or SWS1 [40]–[42].

In this paper, we addressed the function of Rad51 mediators and their relationship in DNA-damage responses. We generated the single Rad51 mediator mutant cells, including *brca2 null*, *sfr1* and *sws1* deficient cells from DT40 chicken cell line [43]. We also disrupted the *BRCA1*, one of the *RAD51* paralogs (*XRCC3*), *RAD52*, *RAD54*, *SWS1*, and *SFR1* in *brca2 null* deficient DT40 cells. The phenotypes of these cells were analyzed to reveal hierarchical relationship of RAD51, BRCA2, and the other RAD51 mediators, where RAD51 is able to operate HR without BRCA2 while BRCA2 is required for the functioning of the other RAD51 mediators. Hence, BRCA2 might serve as a platform to recruit various RAD51 mediators at the appropriate position of DNA-damage sites. Our study sheds light on the functional relationship of RAD51 and every known RAD51 mediators for the first time, and thereby significantly contributes to the development of effective anti-cancer therapies.

## Results

### Loss of SFR1 or SWS1 has a limited impact on DNA–damage responses

**Figure 1:**
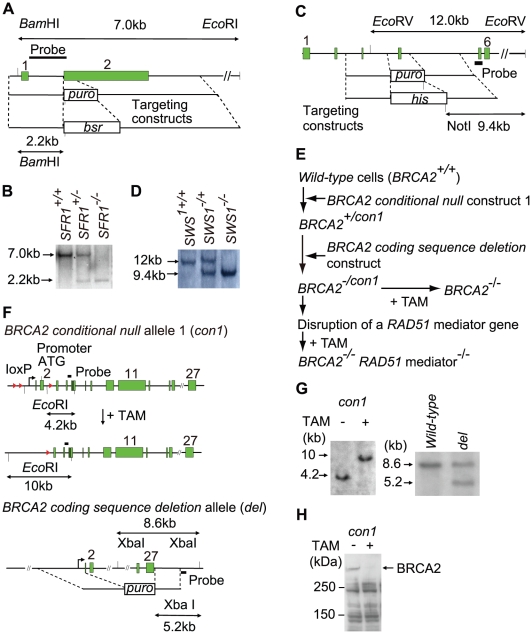
Gene disruption of the *SFR1*, *SWS1*, and *BRCA2* loci. (A) Schematic representation of the endogenous *SFR1* locus and gene-disruption constructs carrying the *puro* or *bsr* selection marker gene. The solid boxes represent exons, and numbers right above boxes represent exon numbers. Relevant *Bam*HI and *Eco*RI sites are indicated. (B) Southern-blot analysis of genomic DNA digested by both *Bam*HI and *Eco*RI was performed using the probe DNA shown in (A). Positions of hybridizing fragments of *wild-type* (WT) and targeted loci are indicated. (C) Schematic representation of the endogenous *SWS1* locus and gene-disruption constructs carrying the *puro* or *his* selection marker gene. Relevant *Eco*RV and NotI sites are indicated. (D) Southern-blot analysis of genomic DNA digested by *Eco*RV and NotI was performed with the probe DNA shown in (C). (E) Experimental methods to generate *BRCA2^−/−^* and *RAD51mediator^−/−^/BRCA2^−/−^* cells. We generated *BRCA2^−/−^* cells from conditional mutant *BRCA2^−/con1^* cells. In the minus allele of the *BRCA2^−/con1^* cells, the whole coding sequence is deleted (hereafter called the *coding sequence deletion* allele). The structures of the *conditional-null* allele-1 (*con1*) is shown in (F). Treatment of *BRCA2^−/con1^* cells with 4-OH tamoxifen (TAM) led to the generation of *BRCA2^−/−^* cells. To generate *RAD51mediator^−/−^/BRCA2^−/−^* cells, we disrupted one of the *RAD51mediator* genes in *BRCA2^−/con1^* cells. Exposure of the resulting *RAD51mediator^−/−^*/*BRCA2^−/con1^* cells to TAM led to the generation of *RAD51mediator^−/−^*/*BRCA2^−/−^* cells. (F) Schematic representation of *BRCA2 conditional-null* allele and the *brca2-null* allele wherein the whole coding sequences are deleted. The *conditional-null* allele-1 (*con1*) shown on top was described previously [33]; the structure of the *coding sequence deletion* allele is shown in the second row. Treatment of the *BRCA2^−/con1^* cells with TAM causes deletion of the promoter and initiation codon. The relevant *Eco*RI sites in the *conditional-null* allele-1, the relevant XbaI sites in the *coding sequence deletion* allele, and the position of the probes used in the Southern-blot analysis (G) are indicated. The solid boxes and arrowheads represent the exons and loxP signals, respectively. (G) Southern-blot analysis of the conditional allele (left) and the other *coding sequence deletion* (−) allele (right) in *BRCA2^−/con1^* cells with (+) or without (−) TAM treatment. Southern-blot analysis of *Eco*RI or XbaI-digested genomic DNA was performed with the probe DNA shown in (F). (H) Western-blot analysis to verify the loss of BRCA2 protein in *BRCA2^−/−^* cells derived from *BRCA2^−/con1^* cells.

**Figure 2:**
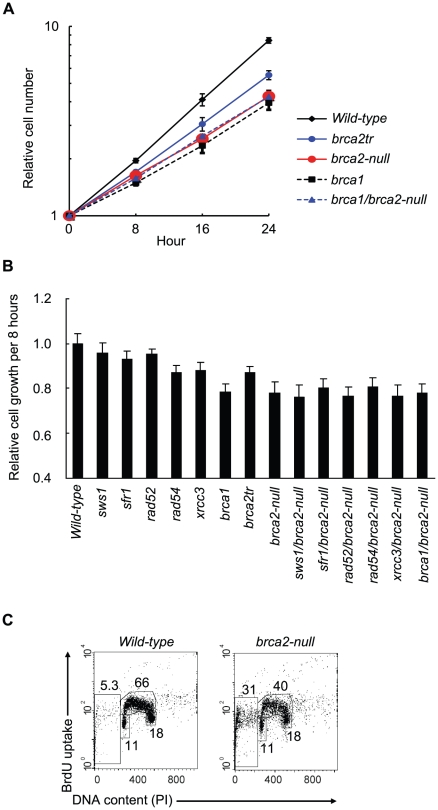
Decreased cellular proliferation in *brca2-null* cells. (A) Growth curve for cells of the indicated genotype. (B) The relative rate of cell growth per 8 hours (a single cell cycle for *wild-type* cells) plotted for cells carrying the indicated genotypes. Each value represents the averaged results from three separate experiments. Error bars represent standard deviation. (C) Cell-cycle distribution of *brca2-null* cells that were pulse-labeled with BrdU for 10 minutes and subsequently stained with FITC-conjugated anti-BrdU antibody (Y-axis, log scale) and propidium iodide (PI) (X-axis, linear scale). The upper gate indicates cells incorporating BrdU (S phase), the lower middle gate indicates G_1_ cells, and the lower-right gate indicates G_2_/M cells. The sub G_1_ fraction (lower-left gate) indicates dead cells. The number in each gate indicates the percentage of gated events.

**Figure 3:**
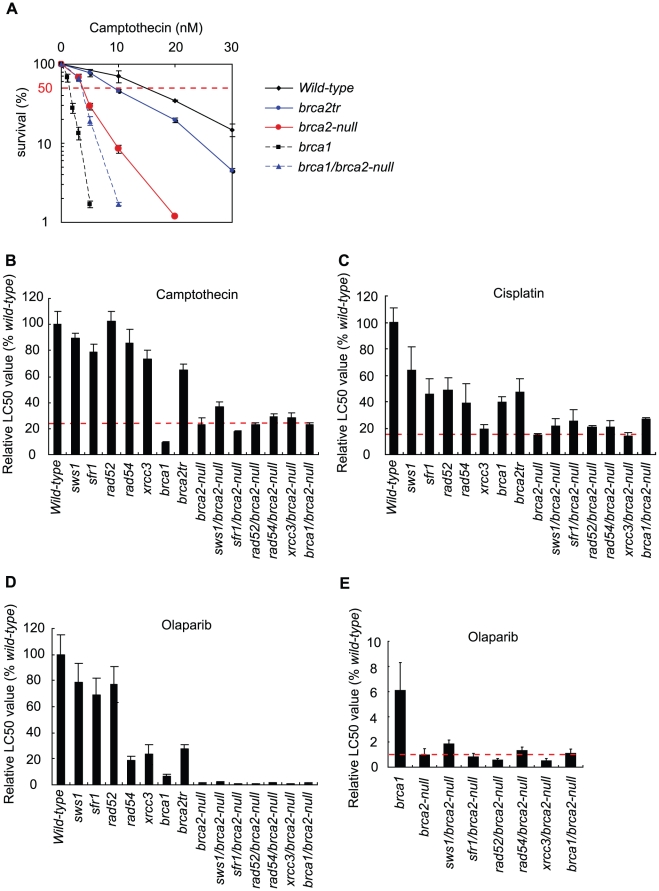
Cellular tolerance to camptothecin, cisplatin, and olaparib. (A) Cells of the indicated genotype were exposed to camptothecin for 72 hours, a period during which *wild-type* cells are able to divide nine times in the absence of exogenous DNA damage. The X-axis represents the concentration of camptothecin and the Y-axis represents the relative number of surviving cells at 72 hours. The vertical dotted lines show LC_50_ values (the concentration of camptothecin that reduces cellular survival to 50% relative to cellular survival without camptothecin treatment). Relative LC_50_ values of camptothecin (B), cisplatin (C), and Poly(ADP-ribose) polymerase inhibitor olaparib (D and E) are shown. Values shown are mean ± SD.

**Figure 4:**
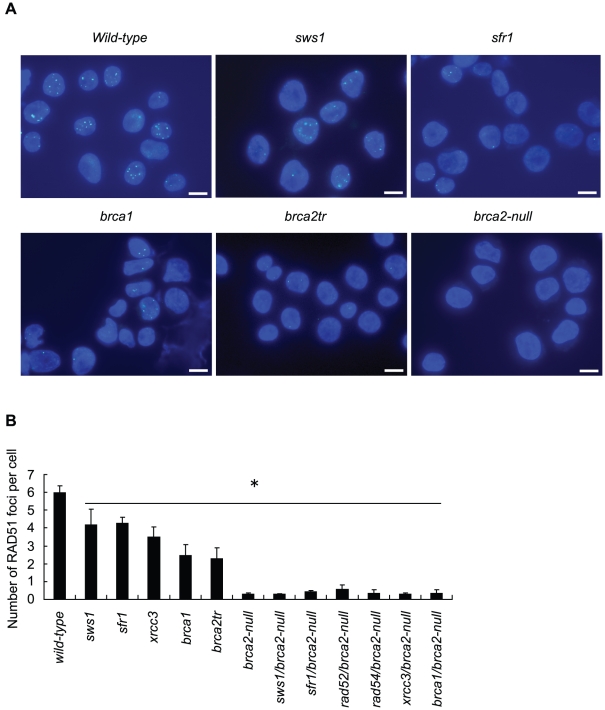
γ-ray–induced Rad51 subnuclear foci in RAD51-mediator mutant cells. (A) Immuno-staining of irradiated *wild-type* and mutant DT40 clones using anti-RAD51 antibody. Cells were fixed 3 hours after irradiation with 4Gy γ-rays. Bar, 10 µm. (B) Quantification of RAD51 foci in individual cells of the indicated genotype. Data shown are the means of three experiments. Error bars indicate standard deviation. Statistical analysis was performed using the *t* test. * *P*<0.01 compared to *wild-type*.

To analyze SFR1 and SWS1, we disrupted the *SFR1* and *SWS1* genes in DT40 cells (Figure 1A–1D). Table 1 summarizes the selection marker genes we used to disrupt genes in this study. The resulting *sfr1* and *sws1* mutant clones proliferated with nearly normal kinetics (Figure 2) and exhibited an increase in cellular sensitivity to cisplatin. The *sfr1* mutant was sensitive also to camptothecin and olaparib (Figure 3). Both mutants showed a slight but significant decrease in ionizing-radiation-induced RAD51 focus formation (Figure 4). We conclude that SFR1 and SWS1 indeed work as RAD51 mediators, though their contribution to HR-dependent repair is less significant than that of BRCA1, BRCA2, and the RAD51 paralogs.

**Table 1:** DT40 mutants used in this study.

Cell line	Selection marker for gene disruption	Reference or source
*sws1*	*puro/his*	This study
*sfr1*	*bsr/puro*	This study
*brca2-null*	*hyg/his*	This study
*rad52*	*bsr/his*	[69]
*rad54*	*neo/his*	[70]
*xrcc3*	*bsr/his*	[71]
*brca1*	*puro/his*	[46]
*brca2tr*	*hyg/his*	[33]
*sws1/brca2-null*	*puro/his*	This study
*sfr1/brca2-null*	*bsr/puro/his*	This study
*rad52/brca2-null*	*hyg/puro/his*	This study
*xrcc3/brca2-null*	*hyg/bsr/puro/his*	This study
*rad54/brca2-null*	*neo/bsr/puro/his*	This study
*brca1/brca2-null*	*hyg/bsr/puro/his*	This study

### The *brca2*-*null* mutant is capable of proliferating

To create *brca2*-*null* cells, we generated compound heterozygous mutant cells (hereafter called *BRCA2^−/con1^* cells) (Figure 1E). The whole coding sequence was deleted in the minus (−) allele of the -/conditional-*null* allele-1 (*-/con1*) genotype of the *BRCA2^−/con1^* cells (*Coding sequence deletion* allele in Figure 1F). We conditionally deleted the *con1* allele of the *BRCA2^−/con1^* cells by adding tamoxifen, which activated the chimeric Cre recombinase [28] and thereby eliminated the promoter and coding sequences, including exons 1 and 2 of the *con1 BRCA2* allelic gene (*BRCA2 conditional-null* allele-1 in Figure 1F). At day two of continuous tamoxifen treatment, the vast majority of cells lost the intact *BRCA2* gene in the conditional allele, and a substantial fraction of cells began to die. To our surprise, we were able to reproducibly establish clonally expanding cells wherein the conditional *BRCA2* allele was deleted (*BRCA2^−/−^* cells, hereafter called *brca2-null* cells) from 10 to 20% of the tamoxifen-treated populations. We verified deletion of the *BRCA2* allele in the established clones by Southern-blot (Figure 1G) and western-blot (Figure 1H) analysis. The ability of the *brca2*-deleted cells to proliferate is in marked contrast to the immediate cell death observed in *rad51*-deleted cells [1]. The plating efficiency of the *brca2-null* clones was around 20%, which is significantly lower than that of the *wild-type* (100%) and *brca2tr* (60%) cells [33].

One obvious concern with this experiment was that expression of the N-terminal-truncated BRCA2 protein in the Cre-mediated deletion lines could allow for residual function. We therefore created a second version of the conditionally inactivated *BRCA2* allele, wherein sequences spanning from the promoter to intron 12 could be eliminated by induction of Cre (*BRCA2* conditional-*null* allele-2 in Figure S1). We exposed the resulting compound heterozygous mutant cells to tamoxifen and confirmed reproducible establishment of *BRCA2*-deleted clones (Text S1). This second *brca*2 *conditional-null* allele supported proliferation with generation times very similar to those of the first version of the *brca2-null* cells (data not shown). The more extensively deleted *brca2* clones showed a phenotype indistinguishable from that of the smaller deletion clones, indicating that both deletions confer the *null* phenotype.

### BRCA2 contributes to genome maintenance to a lesser extent than does RAD51

We assessed the proliferative properties of *brca2*-*null* clones by monitoring their growth curve (Figure 2A and 2B) and cell cycle (Figure 2C). *Wild-type* cells doubled every 8 hours and increased in cell number by 64 times over 48 hours. The *brca2-null* cells increased by 17 times over 48 hours (Figure 2A), calculated as a 1.6-fold increase over 8 hours (1.6^6^ = 17) (Figure 2B). The number of *brca1*, *brca2tr*, and *xrcc3* clones [36] increased 1.6, 1.9, and 1.8 fold, respectively, over eight hours. The reduced growth kinetics of the *brca2-null* cells is partly due to apoptosis in a substantial fraction of the cell population, as evidenced by the accumulation of cells in a sub-G_1_ fraction (Figure 2C).

To understand the cause of this cell death, we measured the number of chromosome breaks in mitotic cells. Forty-six chromosomal aberrations were detectable in 100 mitotic *brca2*-*null* cells, larger than the number of aberrations observed in any other RAD51-mediator mutants (Table 2). We therefore conclude that spontaneously arising DSBs resulted in cell death in a fraction of *brca2-null* cells, thus accounting for the reduced growth kinetics (Figure 2A and 2B). The viability of the *brca2-null* cells reveals that BRCA2 plays a less important role than does RAD51 in the maintenance of genome integrity [1]. The high number of spontaneously arising chromosomal breaks in the *brca2-null* cells (Table 2) shows that BRCA2 plays a more fundamental role in genome maintenance than do any of the other RAD51 mediators.

**Table 2:** Spontaneous chromosomal aberrations.

	Chromatid-type		Chromosome-type			
Cell line	Gaps	Breaks	Gaps	Breaks	Exchange	Total
*wild-type*	1	1	1	0	0	3
*xrcc3*	1	1	4	6	0	12
*rad54*	1.7	0	1.7	0	0.7	4.1
*brca1*	6	10	0	6	2	24
*brca2-null*	16	10	2	18	0	46
*brca2tr*	4	4	3	3	2	16
*brca1/brca2-null*	0	24	2	13	0	39

Spontaneous chromosomal aberration of indicated genotypes. Data are the numbers of aberrations per 100 cells. At least 100 mitotic cells were analyzed for each genotype.

### *brca2-null* cells displayed a stronger phenotype than the *brca1*, *brca2tr*, and *rad51 paralog* mutant clones

We analyzed cellular tolerance to camptothecin, cisplatin, and olaparib by measuring cellular survival at 72 hours (7–9 cell cycles) after continuous exposure to these agents in a liquid medium. We did not use the conventional colony-formation assay for this analysis, because the plating efficiency of the *brca2-null* cells was only 20%, 5-fold lower than that of *wild-type* cells. Figure 3A presents an example of cellular sensitivity to camptothecin, a DNA-damaging agent. Subsequent figures illustrate the sensitivity of each mutant, assessed by LC_50_ values, i.e., the dose that reduces cell survival to 50% relative to the LC_50_ value of *wild-type* cells, which is defined as 100% (Figure 3B–3E).

In the cellular-survival analysis, the *brca2-null* cells showed an increased sensitivity to camptothecin (Figure 3B), cisplatin (Figure 3C), and olaparib (Figure 3D and 3E). Moreover, sensitivity to cisplatin and olaparib was higher with the *brca2*-null cells than for any of the other HR mutant cells, including the *brca1*, *rad52*, *rad54*, and *xrcc3* clones (Figure 3). We therefore conclude that BRCA2 plays a more important role in HR-dependent repair than do the other RAD51 mediators, including BRCA1, RAD52, RAD54, the RAD51 paralogs, SFR1, and SWS1.

The less prominent phenotype of the *brca2tr* cells compared to the *brca2-null* cells indicates that the BRCA2 BRC3-truncated protein retains significant residual HR function. Although *brca1* cells were less sensitive to cisplatin and olaparib than were *brca2-null* cells, the *brca1-null* cells exhibited a slightly higher sensitivity to camptothecin than did the *brca2-null* cells (Figure 3A). The greater contribution of BRCA1 to cellular tolerance to camptothecin might be attributable to the role played by BRCA1 in DNA-damage responses other than HR, such as collaborative action with CtIP to eliminate covalently bound oligo-peptides from DSBs [15].

We next measured the frequency of HR-dependent repair of I-*Sce*1-mediated DSBs in a recombination substrate, SCneo, inserted into the *OVALBUMIN* locus [44], [45] (Table 3). The frequency of HR-dependent DSB repair was calculated as the number of neomycin-resistant (neo^+^) colonies relative to the number of plated cells. The frequency of HR in the *brca2-null*, *brca2tr*, and *brca1* cells was decreased by 1.5×10^4^-, 1.5×10^2^-, and 4.5×10^3^-fold, respectively, compared with *wild-type* cells. We conclude that the *brca2-null* cells retain residual HR activity, which may account for their viability even in the complete absence of BRCA2.

**Table 3:** Double strand break-induced gene conversion frequency.

Cell line	Relative recombination frequency ± SE	Range of neo^+^ colonies per 1×10^7^ transfected cells (range)
*wild-type*	1.0±0.8	1.1∼2.5×10^5^
*xrcc3*	1.3±0.1×10^−1^	1.3∼2.0×10^4^
*brca1*	2.2±0.4×10^−4^	20∼36
*brca2-null*	6.8±0.9×10^−5^	4∼11
*brca2tr*	6.6±4.2×10^−3^	347∼1493
*sws1/brca2-null*	7.1±1.5×10^−5^	8∼15
*sfr1/brca2-null*	5.6±0.2×10^−5^	6∼13
*rad52/brca2-null*	4.4±0.2×10^−5^	5∼10
*xrcc3/brca2-null*	3.0±1.1×10^−5^	2∼13
*rad54/brca2-null*	7.6±1.6×10^−5^	7∼20
*brca1/brca2-null*	1.9±0.5×10^−5^	1∼7

Analysis of HR frequency induced by I-*Sce*1-induced DSBs in an artificial substrate. SCneo-puro was stably integrated into the *OVALBUMIN* locus of indicated genotypes by gene-targeting. 1.0×10^7^ cells of each genotype were transfected with 30 µg of either I-*Sce*1 expression vector or pBluescript KS, and subsequently selected using a neomycin analog (G418). The ratio of recombination frequency was calculated by comparing the number of G418 resistant clones in the mutant cell lines to that in the *wild-type* cell line. Only results obtained using the I-*Sce*1 expression vector are shown.

Since BRCA2 promotes the loading of RAD51 at damage sites, we measured RAD51 focus formation at 3 hours after ionizing radiation. The number of RAD51 foci was reduced but not eliminated in the *brca1* and *brca2tr* clones, compared with *wild-type* cells (Figure 4). These findings are consistent with previous observations [33], [46]. By contrast, we hardly detected any RAD51 focus formation in the *brca2-null* cells. In conclusion, the BRCA2 protein plays a key role in the efficient recruitment of RAD51 to DNA-damage sites, but is not essential for every HR reaction.

### BRCA2 is required for the effective participation of BRCA1, RAD52, SFR1, SWS1, and XRCC3 in HR

The idea that RAD51 carries out HR even without BRCA2 led us to investigate whether or not other RAD51 mediators substitute for BRCA2 in the promotion of RAD51-dependent HR. To this end, we deleted the *BRCA1*, *RAD52*, *SFR1*, *SWS1*, and *XRCC3* genes in the conditional *brca2*-*null* background, then inactivated the *BRCA2* gene by treating the cells with tamoxifen (Figure 1E). We also disrupted the *RAD54* gene in the conditional *brca2*-*null* background (Table 1). The RAD54 protein promotes HR after the assembly of RAD51 at DNA-damage sites [47]. To our surprise, we were able to reproducibly establish all resulting double mutants, although a substantial fraction (∼30%) of the *brca2-null* cells died each cell cycle.

The growth kinetics for the *brca1/brca2-null*, *rad52/brca2-null*, *sfr1/brca2*, *sws1/brca2-null*, and *xrcc3/brca2-null* double-mutant clones was similar to those of the *brca2-null* single mutant (Figure 2). Taking the very severe phenotype of *brca1* cells into account, the viability of the *brca1/brca2-null* cells was surprising. The cloning efficiency of the *brca1/brca2-null* cells was slightly higher than that of the *brca2*-*null* single-mutant cells (30% compared to 20%). Accordingly, the number of spontaneous chromosomal aberrations in the *brca1/brca2-null* cells was consistently slightly lower than that in the *brca2-null* cells (Table 2). In summary, although the loss of either BRCA1 or BRCA2 greatly increased the number of spontaneous chromosomal breaks, inactivation of BRCA1 in the *brca2-null* cells resulted in a slight reduction in the severity of the *brca2* phenotype.

An early study shows *rad52/xrcc3* double-mutant cells are synthetic lethal and exhibit numerous chromosomal breaks [24], whereas we here found that the *brca2/rad52* and *brca2/xrcc3* double-mutant cells were viable. Thus, the synthetic lethality might be attributable to BRCA2 mediated formation of toxic HR intermediates, because the *brca2/xrcc3* cells exhibit spontaneously arising isochromatid breaks, where two sisters are broken at the same site due to defective completion of HR [48]. To test this hypothesis, we conditionally inactivated the *BRCA2* gene in the *rad52/xrcc3* cells (Text S1). We found that the inactivation of the *BRCA2* gene indeed rescued the *rad52/xrcc3* cells (Figure S2). This observation indicates that the synthetic lethality of the *rad52/xrcc3* cells does not argue against the idea that the functioning of RAD52 and XRCC3 depends on BRCA2. Likewise, the formation of toxic HR intermediates might explain the apparent discrepancy between the viability of *rad52/brca2-null* DT40 cells and the mortality caused by shRNA mediated depletion of RAD52 in *brca2* deficient mammalian cells [49], as the latter cells express a residual amount of RAD52 and truncated BRCA2 proteins perhaps leading to the formation of toxic HR intermediates.

**Figure 5:**
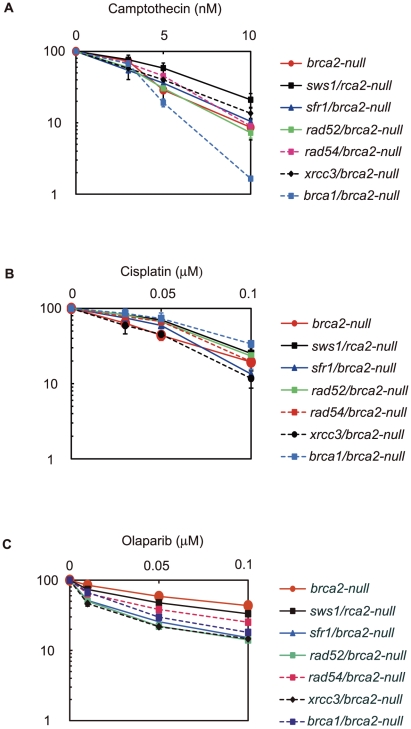
Effect of *brca2* deletion on *sws1-*, *sfr1-*, *rad52-*, *rad54-*, *xrcc3-*, and *brca1-*deficient cells. Cellular sensitivities to the indicated DNA damaging agents were analyzed using the same method as in Figure 3. The LC_50_ values are shown in Figure 3B–3E.

We next measured the sensitivity of the *brca1/brca2-null*, *rad52/brca2-null*, *rad54/brca2-null*, *sfr1/brca2-null*, *sws1/brca2-null*, and *xrcc3/brca2-null* double-mutant clones to camptothecin, cisplatin, and olaparib (Figure 3 and Figure 5). Remarkably, inactivation of any gene did not increase cellular sensitivity to the three damaging agents by more than two-fold. This observation indicates that the contribution made by BRCA1, the RAD51 paralogs, RAD52, RAD54, SFR1, and SWS1 to HR depends mostly on BRCA2. Interestingly, the loss of BRCA1, SFR1, and SWS1 somewhat increased the cellular tolerance of the *brca2-null* cells to cisplatin. Similarly, the loss of SWS1 increased the cellular tolerance of the *brca2-null* cells to camptothecin and olaparib. This increased tolerance was not accompanied by the upregulation of RAD51 focus formation (data not shown). We therefore suggest that, in the absence of BRCA2, SWS1 has a moderately antagonistic effect on HR-dependent repair. By contrast, the loss of RAD52 and XRCC3 significantly increased the cellular sensitivity of the *brca2-null* cells to olaparib. In summary, BRCA2 is required for all the analyzed RAD51 mediators to function, and the functional relationships between BRCA2 and the other RAD51 mediators in HR-mediated repair differ slightly depending on the type of DNA damage.

## Discussion

In this study, we established *brca2-null* cells as well as cells deficient in each of the RAD51 mediators. We show that BRCA2 plays a more important role in the promotion of both RAD51 polymerization at DNA-damage sites and HR-dependent repair than does any other RAD51 mediator, including BRCA1, the RAD51 paralogs, RAD52, SFR1, and SWS1. The ability of *brac2-null* cells to proliferate is in marked contrast with the immediate cell death that occurs upon depletion of RAD51 [1]. Therefore, RAD51 is able to perform HR even in the absence of BRCA2. To explore the question of which RAD51 mediators might substitute for BRCA2 in the promotion of RAD51-dependent HR repair, we inactivated the RAD51 mediators in *brca2-null* cells. Loss of any one of the other RAD51 mediators did not further reduce the viability of *brca2-null* cells. In a related study, we also found that the *brca2-null* mutant and the *palb2/brca2-null* double mutant showed the same phenotype with respect to both spontaneous chromosomal aberrations and increased sensitivity to DNA-damaging agents (manuscript in preparation). Thus we conclude that BRCA1, PALB2, the RAD51 paralogs, RAD52, SFR1, and SWS1 all require BRCA2 to contribute to HR.

### BRCA2 plays a major role in the recruitment of RAD51 to DNA–damage sites, but is not essential for every HR reaction

Data on *Ustilago maydis* [50] and *Arabidopsis thaliana* [51] suggest that BRCA2 might be essential for RAD51 to function in any HR reaction. However, we here report that RAD51 can form HR products even in *brca2-null* cells, indicating that RAD51 plays a more important role than BRCA2 in HR. This hierarchy between RAD51 and BRCA2 is supported by previous reports of experiments with mice, as *rad51 null* embryos died earlier (∼E6.5) than did *BRCA2 null* (∼E8.5) embryos [52], [53]. The viability of *brca2-null* DT40 cells is consistent with the clonal expansion of BRCA2-deficient cells derived from mammary epithelial lineage-specific or T cell lineage-specific BRCA2-*null*-deficient mice [54], [55]. Adding to these findings, we here show solid evidence that vertebrate RAD51 is capable of functioning in the absence of BRCA2.

### Collaboration between BRCA1 and BRCA2 is required for efficient HR

The phenotypic analysis of *brca1*, *brca2-null*, and *brca1/brca2-null* clones, combining with the previous study of *rad51-null* cells, reveals the functional relationship described as follows. The capability of HR was dramatically diminished when either BRCA1 or BRCA2 was absent, indicating that the collaboration of BRCA2 and BRCA1 is required for efficient HR events. *brca2-null* cells exhibited more prominent defects in HR than did *brca1-null* cells, indicating that BRCA2 can function in HR independently of BRCA1. Moreover, BRCA2′s contribution to the repair of cisplatin-induced interstrand crosslinks is more significant than BRCA1, which is likely attributable to the fact that BRCA2, but not BRCA1, functions in the Fanconi anemia repair pathway [56]. BRCA1 has additional functions other than in HR, such as mediating the damage checkpoint and processing DSBs [15], [57]. The fact that *rad51-null* cells have a considerably stronger phenotype than *brca2-null* cells indicates that RAD51 could still perform HR-dependent repair in *brca2-null* cells.

The phenotypic similarities between the *brca2-null* and the *brca1/brca2-null* clones indicate that BRCA1 contributes to HR by collaborating with BRCA2. Presumably, the two BRCA proteins form a functional unit and collaborate intimately to load RAD51 at damage sites. This idea is supported by the fact that BRCA1 physically associates with BRCA2 through the PALB2 protein [58]. However, this idea is challenged by recent studies that suggest that BRCA1 plays a role in the resection of DSBs [14], [59]. One possible scenario is that the complex formation of BRCA1 and BRCA2 may allow for close collaboration between the BRCA1-dependent resection of DSBs and the subsequent loading of RAD51 on the resulting 3′ overhang. Such an interaction interface might be shared by the E. coli RecBCD complex, which serves as the DSB resection complex and also interacts directly with RecA following chi site recognition [60]. In summary, the phenotypic analysis of *brca1*, *brca2-null*, and *beca1/brca2-null* DT40 clones demonstrates that BRCA1 controls RAD51 in HR, mainly through collaboration with BRCA2.

### BRCA2 is required for BRCA1, PALB-2, the RAD51 paralogs, SFR1, and SWS1 to promote HR

**Figure 6:**
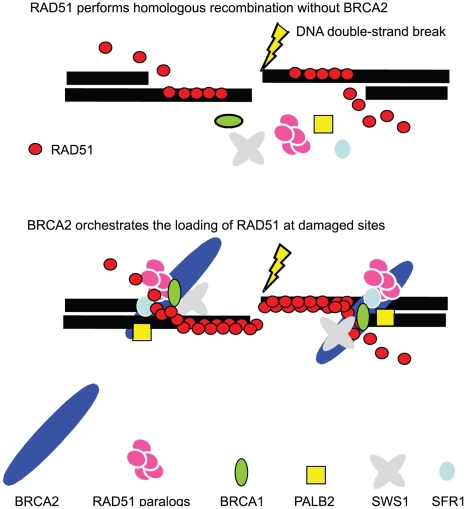
Model of BRCA2 dependent regulation of various RAD51 mediators at DNA–damage sites. In the absence of BRCA2, RAD51 polymer assembles at DNA damage sites (top), while BRCA2 significantly promotes the polymerization of RAD51 (bottom). This promotion is attributable to the requirement of BRCA2 for the appropriate localization of other RAD51 mediators at DNA damage sites. Accordingly, the loss of BRCA2 may result in the abnormal functioning of some RAD51 mediators, for example, SWS1. This scenario explains why the loss of SWS1 increased cellular tolerance to camptothecin, cisplatin, and olaparib only when BRCA2 was absent (Figure 3).

Our study reveals that *rad52/brca2-null*, *sfr1/brca2-null*, *sws1/brca2-null*, and *xrcc3/brca2-null* clones exhibit a phenotype very similar to that of *brca2-null* cells (Figure 5). In a separate study, we conformed phenotypic similarity between *brca2-null* and *palb2/brca2-null* clones (data not shown). We therefore suggest that, like BRCA1, PALB2, the RAD51 paralogs, RAD52, SFR1, and SWS1 are also able to participate in HR, mostly depending on BRCA2. One possible scenario is that BRCA2 is recruited to DNA-damage sites through PALB2 or by directly interacting with the junction between the duplex DNA and the single-strand sequences [61]. BRCA2 might thus serve as a platform to recruit various RAD51 mediators to the appropriate positions of DNA-damage sites (Figure 6).

### Applications for clinical research

DT40 is a unique cell line that offers a panel of DNA-repair-deficient isogenic mutants derived from a stable parental line. DT40 cells have several characteristics that affect cellular responses to anti-cancer agents. First, DT40 appears, for unknown reasons, to possess a significantly higher HR efficiency than any mammalian cell line [43]. The efficient HR in DT40 cells is prominent particularly in HR between diverged homologous sequences such as Immunoglobulin V gene diversification [62] and gene targeting, where the selection marker genes of gene-disruption constructs may interfere with HR as heterologous sequences. Second, like many cancer cells, DT40 lacks the functional p53, and as a result has no G_1_/S damage checkpoint [63]. In addition, 70% of the DT40 cell cycle takes place in the S phase. Thus, DNA damage at any phase of the cell cycle may have a direct impact on DNA replication. These characteristics, specific to DT40, suggest that a defect in DNA repair associated with DNA replication, including HR-mediated DNA repair, may display a more prominent phenotype in DT40 cells than in other cell lines that have a longer G_1_ phase and/or a normal G_1_/S checkpoint. Bearing this in mind, DT40 is revealed as a unique and valuable tool and has been used extensively to explore the role of individual HR factors responsible for cancer therapy.

## Materials and Methods

### Cell culture and DNA transfection

Cells were aquired and cultured as described previously [1], [43]. All mutants were isolated from single colonies. DNA transfection and selection were performed as described previously [43], [64]. Details of the cell lines used in this study are shown in Table 1.

### Generation of *SFR1^−/−^* DT40 cells

To disrupt the *SFR1* gene, we generated *SFR1-puro* and *SFR1-bsr* disruption constructs by combining two genomic PCR products with the puro- or bsr-selection-marker cassette. Genomic DNA sequences were amplified using the 5′-CCCGGTACTGAGGGGTGCGATTGCTTGCAGG-3′ and 5′-CCCTTAGAGTTGCACTCATTGGCTAAAG-3′ primers for the upstream arm, and the 5′-GGCTCAAACTGGTCAAGATGTACCGATCTAAGG-3′ and 5′-CCACCAGCATCCACTAAAGGGCAAGGAACG-3′ primers for the downstream arm. Amplified PCR products were cloned into pCR2.1-TOPO vector (Invitrogen). The 1.7 kb fragment of the upstream arm was cloned into the KpnI site of pCR2.1 containing the 3.0 kb downstream arm. Marker-gene cassettes were inserted at the *Bam*HI site of the resulting plasmid.

To generate *SFR1^−/−^* cells, *SFR1-puro* and *SFR1-bsr* disruption constructs lineralized with NotI were transfected sequentially by electroporation (Bio-Rad). The genomic DNA of the transfectants was digested with both *Bam*HI and *Eco*RI, and gene-targeting events were confirmed by Southern blot analysis. The probe was prepared from a PCR-amplification of DT40 genomic DNA using primers 5′-GAACAGCACCACGCAATTCA-3′ and 5′-CCTTAGAGTTGCACTCATTGG-3′.

### Cloning of *SFR1* cDNA

Chicken *SFR1* cDNA was isolated by PCR amplification of the primary cDNAs using the 5′-GTTGAGATGGAGGAAGCAGCGTGTGGTAAA-3′ and 5′-CACCACTCAATTCCACTTCAAAGAG-3′ primers. The gene bank accession number of the chicken *SFR1* gene is XM-001234167.

### Generation of *SWS1^−/−^* DT40 cells

To disrupt the *SWS1* gene, we generated *SWS1* gene-disruption constructs containing the 2.6 kb upstream and the 3.0 kb downstream genomic fragments. The 2.6 kb fragment was PCR-amplified using the 5′-ggggacaactttgtatagaaaagttgTTCTTACGTCACTCCAGAAGAACA-3′ and 5′-ggggactgcttttttgtacaaacttgCCAAGTCTGTGAATCGCAGAAGCA-3′ primers. The 3.0 kb fragment was PCR-amplified using the 5′-ggggacagctttcttgtacaaagtggAATTCCAAGCAGTTCCACATCTCT-3′ and 5′-ggggacaactttgtataataaagttgGTATGGCTCCTGTCAGGTTAGAGT-3′ primers. Note that the underlined sequences denote the recognition sequences in the Gateway system (Invitrogen). Using the MultiSite Gateway system with pENTR-lox-his, pENTR-lox-puro and pDEST-DTA-MLS [65], a floxed *his* or *puro* gene was inserted between the upstream and downstream arms on a plasmid carrying a diphtheria toxin A (DT-A) gene, thus yielding the two targeting vectors, *SWS1-his/loxP* and *SWS1-puro/loxP*.

To generate *SWS1^−/−^* cells, *SWS1-his/loxP* and *SWS1-puro/loxP* gene-disruption constructs linearized with AscI were transfected sequentially into DT40 cells (Bio-Rad). The genomic DNA of the transfectants was digested with both *Eco*RV and NotI, and gene-targeting events were confirmed by Southern-blot analysis. The probe was prepared by PCR-amplification of chicken genomic DNA using the 5′-GCTCGCAGGAACACAACTCCTT-3′ and 5′-GTACAGGAGTGTTTCTCTGCGG-3′ primers.

### Cloning of *SWS1* cDNA

The gene bank accession numbers for the human and chicken *Sws1* genes are XP-058899 and XP-415841, respectively. RT-PCR of DT40 transcripts was done using the 5′-CGCGTCGACATGGATAGCACCTTACCAGCT-3′ and 5′-CGCGGATCCTCATCCTTCATCCTCTTCCTC-3′ primers.

### Generation of *BRCA2^−/−^* DT40 cells

The *brca2-null* mutant cells were generated as follows (Figure 1). We inserted conditional *brca2 heterozygous* cells (*BRCA2^+/con1^*) harboring two loxP signals into the other allele upstream of the promoter and downstream of exon 2. Construction of the *BRCA2* conditional-null targeting vector was carried out as described previously [33]. To delete the intact allele of the *BRCA2^+/con1^* cells, we constructed a targeting vector to delete all exons of the *BRCA2* gene. The ∼6. kb and ∼3.5 kb fragments at the *BRCA2* locus [66] were amplified from DT40 genomic DNA by using the 5′-CCGCTCGAGTTTTGTTAGTTGTGAGATGTG-3′ and 5′-TTATCGGGGCTTTGTCAGCTTTAGCTTCTC-3′ primers and the 5′-CGGAGTTGAATAATGGTACATTTCTGGCAC-3′ and 5′-GTTGAATTTGAAACTGGCTGAACAGAAGAG-3′ primers, respectively. Both fragments were cloned into TOPO-pCRXL cloning vector (Invitrogen, Carlsbad, California) to make the topo/6.0 kb and topo/3.5 kb vectors. The ∼5.2 kb NotI fragment from the topo/6.0 kb vector was inserted into the NotI site in the multicloning site of the topo/3.5 kb vector, resulting in the pUpper/Lower vector. Finally, a loxP-flanked puro-resistance cassette was inserted into the BglII site in the pUpper/Lower vector. The resulting targeting construct was transfected into the *BRCA2^+/con1^* cells followed by selection with puromycin. The genomic DNA of the transfectants was digested with XbaI, and gene-targeting events were confirmed by Southern-blot analysis with a probe that was amplified from genomic DNA using the 5′-ATCCATGTCACTGTTGACATCCTGACTGCC-3′ and 5′-AGATACAAACCCAATGGGAAGCCAGGTGTG-3′ primers. The bands detected by the probe were 8.6 kb from the *wild-type* allele and 5.2 kb from the targeted allele.

Upon exposure of the *BRCA2^−/con1^* cells to tamoxifen, an estrogen antagonist, nucleotide sequences, including promoter and coding sequences encoding the initiation codon to the 67th amino acid, were excised by a chimeric Cre recombinase fused to the estrogen-receptor ligand-binding domain [24], leading to the complete disruption of the *BRCA2* gene.

### Disruption of individual HR genes in *BRCA2^−/−^* DT40 cells

To disrupt *RAD51* mediator genes in *BRCA2^−/−^* DT40 cells, we disrupted each gene in the *BRCA2^−/con1^* cells (Table 1). We exposed the resulting *RAD51 mediator^−/−^*/*BRCA2^−/con1^* cells to tamoxifen and isolated the *RAD51 mediator^−/−^*/*BRCA2^−/−^* cells.

### Western blot analysis

Western blotting was performed as previously described [33]. The rabbit polyclonal primary antibody, which recognizes the N-terminal 203 amino acids of chicken BRCA2, was diluted 1∶100 with blocking buffer. The anti-rabbit IgG HRP conjugated antibody was diluted 1∶5000 with blocking buffer.

### Flow-cytometric analysis

To measure growth kinetics, cells were counted daily using flow-cytometric analysis, as described previously [7]. To measure cell-cycle distribution, cells (5×10^5^/ml) were labeled for 10 minutes with 20 µmol/L 5-bromo-2′-deoxyuridine (BrdU) and subsequently harvested. Harvested cells were fixed and analyzed as previously described [7].

### Measurement of cellular sensitivity to camptothecin, cisplatin, and olaparib in liquid culture

To measure cellular survival, cells (1.5×10^3^–1.5×10^4^) were incubated in 1 ml culture medium per well containing various concentrations of the DNA-damaging agents. At 72 hours, the ATP in the cellular lysates was measured to assess the number of live cells. The camptothecin (TopoGen Inc., Colombus, OH) and olaparib (AstraZeneca) were diluted with DMSO, and the cisplatin (Nihonkayaku, Tokyo, Japan) was diluted with PBS. To measure the sensitivity of the DT40 cell lines to these agents, cells were continuously exposed to various concentrations of the drug and the number of cells was measured at 72 hours. At least three independent experiments were carried out. Sensitivity was calculated by dividing the number of cells treated with the drug by the number of untreated cells [8].

### Measurement of ATP to assess cellular sensitivity to DNA damaging agents

To assess cell numbers after treatment with the genotoxic reagents, we measured the amount of ATP in the whole cell lysate [67].

### Visualization of RAD51 foci

Cells were harvested at 3 hours after gamma irradiation. Cells were spun onto slides using a Shandon Cytospin 3 centrifuge (Shandon, Pittsburgh, Pa.). Staining and visualization of RAD51 foci were carried out as previously described [34] using rabbit polyclonal antibody, which recognizes human RAD51, at a dilution of 1∶500 (Calbiochem, San Diego, CA San Diego, CA), and Alexa Fluor 488 goat anti-human IgG antibody at a dilution of 1∶1000 (Molecular Probes Inc., Eugene, OR [34]).

### Analysis of chromosomal aberrations

Measurement of chromosomal aberrations was performed as described previously [68].

### Measurement of HR frequencies for I-*Sce*1–induced DSB repair

Measurement of recombination frequencies for I-*Sce*I-induced DSB repair was performed as described previously [34], [44]. Modified SCneo was inserted into the previously described *OVALBUMIN* gene construct and targeted into the *OVALBUMIN* locus in *wild-type*, *xrcc3*, *brca1*, *brca2*, and *brca2tr* DT40 clones. For transient transfections, 1×10^7^ cells were suspended in 0.5 ml of phosphate-buffered saline, mixed with 30 µg of I-*Sce*I expression vector (pCBASce) or pBluescript KS without linearization, and electroporated at 250 V, 960 microfarads. At 24 hours after electroporation, the cells were plated in 96-well plates with or without 2.0 mg/ml neomycin analog (G418). The cells were grown for 7 to 10 days, after which formed colonies were counted. HR frequency was calculated by dividing the number of neomycin-resistant colonies by the number of plated cells.

### Statistical analysis

Survival data were log-transformed giving approximate normality. Analysis of covariance (ANCOVA) was used to test for differences in the linear dose-response curves between *wild-type* and a series of mutant cells or *brca2-null* cells and a series of double-knockout mutant cells. Viability of the DT40 cells was estimated using regressing curves. Regression-curve equations were used to calculate LC_50_ (50% lethal concentration) values. Relative LC_50_ values were normalized according to the LC_50_ value of the parental *wild-type* cells.

## Supporting Information

Figure S1 Generation of *BRCA2^−/−^* (ver.2) cells. (A) Experimental methods to generate *BRCA2^−/−^* (ver.2) cells. (B) Schematic representation of the *BRCA2 conditional-null* allele-2. The *conditional-null* allele-2 (*con2*) was generated by targeting the *his* selection-marker gene flanked by two loxP signals (ploxP-his) in intron 12 of the *BRCA2* conditional allele 1. Treatment of the *BRCA2^−/con2^* cells causes deletion from the promoter to exon 11, encoding all BRC motifs. The relevant XhoI sites in the *conditional-null* allele-1 and the position of the probe used in the Southern-blot analysis are indicated. The solid boxes and arrowheads represent the exons and loxP signals, respectively.
(EPS)

Figure S2 Proliferation and spontaneous chromosomal aberrations of brca2/rad52/xrcc3 triple knockout cells. (A) Growth kinetics of the indicated cell cultures in the absence (right) and presence (left) of TAM. Each value represents the averaged results from two separate clones. (B) Cell viability was assessed by flow cytometric analysis of PI uptake and forward scatter (FSC) representing the cell size after tamoxifen (TAM) treatment 4 days. A fixed number of plastic beads was added before flow cytometric analysis to calibrate cell number. Cells falling in the R1 and R2 gates identify dead and viable cells, respectively, and numbers given show their percentages. (C) Spontaneous chromosomal aberrations in cells with indicated genotype after tamoxifen (TAM) treatment 6 days. The data shown in the histogram indicate the types and numbers of chromosomal breaks in 50 analyzed mitotic cells. Two breaks at the same site of both sister chromatids are defined as isochromatid breaks, while breaks at either sister chromatid are chromatid breaks.
(EPS)

Text S1 Supplementary materials and methods.
(DOC)
