# Supplementary material for: The Epistatic Relationship between BRCA2 and the Other RAD51 Mediators in Homologous Recombination
Source: PLoS Genet. 2011 Jul 14;7(7):e1002148. doi: 10.1371/journal.pgen.1002148 (PMC3136442; doi:10.1371/journal.pgen.1002148)
Supplement: Text S1 — Supplementary materials and methods. (DOC) [file pgen.1002148.s003.doc]

**Text S1 and S2**

**Generation of the second version of the *brca2-null* cells**

To generate the second version of the *brca2-null* cells, we constructed a conditional null construct 2 vector with a floxed selection marker (*hisD*). The short-arm and long-arm fragments at the *BRCA2* locus were amplified from DT40 genomic DNA by using the primer pairs 5’‑GGGGTACCATAGACTCGTTTAGCTGCACAAGTTCAG‑3’ and 5’‑CCCTCGAGTTGAAATAAGGTGTTTAAGAACAGTGGT‑3’, and 5’‑GCTCTAGAAATGATTTTGTAACGAGGTGTTTGAGT‑3’ and 5’‑GCTCTAGATTCAGAGAAGACTATTTTCTAAGGCAC‑3’, respectively. Both fragments were cloned into pBluescript II (Invitrogen, Carlsbad, Calif.), resulting in pMY523. Finally, the floxed histidine resistance cassette was inserted into the *Bam*HI site in the pMY523 vector. The vector was inserted into an intron between exons 12 and 13 of the *BRCA2* gene. The genomic DNA of *brca2^+/con1^* cells transfected with the conditional null construct 2 vector was digested with XhoI, and gene-targeting events were confirmed by Southern blot analysis. The size of the bands was more than 25 kb from the *wild-type* allele and 9.4 kb from the targeted allele. The other intact allele was deleted as in version 1 for the *brca2-null* cells above. After confirming the genotype of the *brca2^-/con2^* cells, they were treated with TAM, resulting in the generation of the second version of *brca2-null* cells.

**Generation of *BRCA2^-/con1^*/*rad52^-/-^/xrcc3^-/con^* cells**

To conditionally inactivate the *BRCA2* gene in the *rad52^-/-^/xrcc3^-/con^* cells, we sequentially transfected the *conditional-null* allele-1 (con1) construct (Figure 1) and then a targeting vector to delete all exons of the *BRCA2* gene (Figure 1). Since we failed to delete the + allele of *BRCA2^+/con1^*/*rad52^-/-^/xrcc3^-/con^* cells, we generated another construct called the deletion construct 2 (del2), to completely inactivate the remaining *BRCA2* allelic gene. To this end, we amplified the 8.1 kb fragment covering from the 1^st^ to 9^th^ exons of *BRCA2* gene from DT40 genomic DNA by using the 5’- CTCAGGTTTGCCAACAAGTGTTCCAGTGAG-3’ and 5’- TTACCGTCTCTGGTATGGACAGCAGACTTG-3’ primers, and then cloned into the Bluescript II KS+ vector. The 1.1kb *Bam*HI fragment covering from the 4^th^ intron to 7^th^ exon was replaced by a *Bam*HI fragment of *Eco-gpt* marker gene. We linearized the resulting targeting construct with *Pvu*I and transfected into the *BRCA2^+/con1^*/*rad52^-/-^/xrcc3^-/con^* cells followed by selection with 25μg/ml mycophenolic acid (Calbiochem). To identify clones having gene targeting events, we digested the genomic DNA of stable transfectants with *Eco*RI, and subjected to Southern-blot analysis with a probe that was amplified from genomic DNA using the 5’‑ GAGACGGTAAAACTAAATGCTAGAGATGAC ‑3’ and 5’‑ TTAATAGCCATAGGTAGTAGTCTTACTCCC ‑3’ primers. Note the bands detected by the Southern-blot analysis were 4.1kb from the *wild-type* allele and 12 kb from the targeted allele in *BRCA2^+/con1^*/*rad52^-/-^/xrcc3^-/con^* cells. Then, the same Southern-blot filter was re-hybridized with the probe for con1 (Figure 1) to check which allele (either *wild-type* or conditionally disrupted allele) was disrupted. This Southern blot analysis of genomic DNA derived from *BRCA2^+/con1^*/*rad52^-/-^/xrcc3^-/con^* cells shows a 6.9 band derived from the *wild-type* *BRCA2* allele and a 4.2 kb band derived from the conditionally disrupted (*con1*) allele. Accordingly, we identified *BRCA2^-/con1^*/*rad52^-/-^/xrcc3^-/con^* cells by analyzing the disappearance of the 6.9kb band.
